# Supplementary material for: Food By-Product Valorization by Using Plant-Based Coagulants Combined with AOPs for Agro-Industrial Wastewater Treatment
Source: Int J Environ Res Public Health. 2022 Mar 31;19(7):4134. doi: 10.3390/ijerph19074134 (PMC8998984; doi:10.3390/ijerph19074134)
Supplement: Supplementary file 1 [file ijerph-19-04134-s001.zip › ijerph-1619174-supplementary.pdf]

International Journal of
Environmental Research and Public Health

MDPI

Article

Food by-Products Valorization as Plant-Based Coagulants Combined with AOPs for Agro-Industrial Wastewater Treatment

Rita Beltrão Martins [1,2], Nuno Jorge [2,3], Marco S. Lucas [2], Anabela Raymundo [4], Ana I.R.N.A. Barros [1] and José A. Peres [2,*]

1 Centre for the Research and Technology of Agro-Environmental and Biological Sciences (CITAB)/Inov4Agro (Institute for Innovation, Capacity Building, and Sustainability of Agri-Food Production), University of Trás-os-Montes and Alto Douro (UTAD), 5000-801 Vila Real, Portugal; ritabeltraomartins@icloud.com (R.B.M.); abarros@utad.pt (A.I.R.N.A.B.)
2 Centro de Química de Vila Real (CQVR), Departamento de Química, Universidade de Trás-os-Montes e Alto Douro (UTAD), Quinta de Prados, 5000-801, Vila Real, Portugal, mlucas@utad.pt (M.S.L.); jperes@utad.pt (J.A.P.)
3 Escuela Internacional de Doctorado (EIDO), Campus da Auga, Campus Universitário de Ourense, Universidade de Vigo, As Lagoas, 32004, Ourense, Spain, njorge@uvigo.es (N.J.)
4 LEAF—Linking Landscape, Environment, Agriculture and Food, Instituto Superior de Agronomia, Universidade de Lisboa, Tapada da Ajuda, 1349-017 Lisbon, Portugal, anabraymundo@isa.ulisboa.pt (A.R.)
* Correspondence: jperes@utad.pt

Citation: Martins, R.B.; Jorge, N.; Lucas, M.S.; Raymundo, A.; Barros, A.I.R.N.A.; Peres, J.A. Food By-Product Valorization by Using Plant-Based Coagulants Combined with AOPs for Agro-Industrial Wastewater Treatment. *Int. J. Environ. Res. Public Health* **2022**, *19*, 4134. https://doi.org/10.3390/ijerph19074134

Academic Editor: Elena Rada

Received: 15 February 2022
Accepted: 28 March 2022
Published: 31 March 2022

Publisher's Note: MDPI stays neutral with regard to jurisdictional claims in published maps and institutional affiliations.

Copyright: © 2022 by the authors. Submitted for possible open access publication under the terms and conditions of the Creative Commons Attribution (CC BY) license (https://creativecommons.org/licenses/by/4.0/).

Table S1. Consumption of hydrogen peroxide (% H_2O_2 removal) along the photo-Fenton process, under different experimental conditions (pH, H_2O_2, Fe^{2+} and I_{UV}).

**Time**	**pH**				**$[H_2O_2]_0$ (mM)**				**$[Fe^{2+}]$ (mM)**				**I_{UV} (W m^{-2})**			
**(min)**	**3.0**	**4.0**	**6.0**	**7.0**	**9.7**	**19.4**	**38.8**	**77.6**	**0.5**	**1.0**	**2.5**	**5.0**	**0.0**	**5.2**	**18.3**	**32.7**
0	0.0	0.0	0.0	0.0	0.0	0.0	0.0	0.0	0.0	0.0	0.0	0.0	0.0	0.0	0.0	0.0
5	2.0	0.0	2.2	0.0	7.1	0.0	2.0	12.1	2.0	2.0	2.9	11.2	0.0	4.0	3.7	2.0
15	17.8	18.8	18.2	0.0	17.7	21.5	17.8	11.9	2.0	17.8	21.6	23.0	0.0	4.0	3.7	17.8
30	23.0	22.0	24.8	17.0	31.2	34.5	23.0	14.2	15.1	23.0	33.2	34.7	0.0	4.0	3.7	23.0
60	36.8	36.8	36.9	36.8	30.9	35.5	36.8	34.0	26.8	36.8	36.4	36.2	0.0	16.8	36.5	36.8
90	49.0	41.3	44.6	43.4	44.7	45.3	49.0	30.8	28.5	49.0	38.5	44.7	0.0	28.2	39.6	49.0

Table S2. Iron residual concentrations (mg L^{-1}) along the photo-Fenton processes, under different operational conditions (pH, H_2O_2, Fe^{2+} and I_{UV}).

**Time**	**pH**				**$[H_2O_2]_0$ (mM)**				**$[Fe^{2+}]$ (mM)**				**I_{UV} (W m^{-2})**			
**(min)**	**3.0**	**4.0**	**6.0**	**7.0**	**9.7**	**19.4**	**38.8**	**77.6**	**0.5**	**1.0**	**2.5**	**5.0**	**0.0**	**5.2**	**18.3**	**32.7**
**0**	0.09	0.09	0.09	0.09	0.09	0.09	0.09	0.09	0.09	0.09	0.09	0.09	0.09	0.09	0.09	0.09
**5**	48.87	55.65	52.82	35.89	52.82	58.47	48.87	48.31	26.29	48.87	106.45	129.03	55.08	51.13	57.90	48.87
**15**	56.21	58.47	58.47	43.79	55.65	60.73	56.21	48.87	26.29	56.21	104.20	114.92	53.95	55.08	56.77	56.21
**30**	59.03	55.65	33.06	7.10	57.90	55.08	59.03	15.56	25.16	59.03	52.82	74.28	50.00	57.90	57.34	59.03
**60**	55.65	34.19	27.98	7.66	54.52	57.34	55.65	6.53	7.10	55.65	63.55	92.90	51.13	60.16	31.94	55.65
**90**	7.66	5.40	4.84	1.45	51.69	8.23	7.66	2.02	2.02	7.66	4.27	30.81	54.52	56.21	13.31	7.66

Table S3. Pseudo-first order kinetic rate and half-life along the photo-Fenton processes, under diferent operational conditions (pH, H_2O_2, Fe^{2+} and I_{UV}).

**pH**	**$[H_2O_2]$** (mM)	**$[Fe^{2+}]$** (mM)	**I_{UV}** (Wm^{-2})	**k** (min^{-1})	**R^2**	**$t_{1/2}$** (min)
**Variation of pH**						
3.0	38.8	1.0	32.7	0.025±0.0018 a	0.979	27.9±0.436 a
4.0	38.8	1.0	32.7	0.016±0.0012 b	0.978	42.2±1.417 b
6.0	38.8	1.0	32.7	0.020±0.0017 c	0.971	33.8±0.838 c
7.0	38.8	1.0	32.7	0.023±0.0012 d	0.989	30.3±0.216 d
**Variation of H_2O_2 concentration**						
3.0	9.7	1.0	32.7	0.007±0.0001 a	0.968	100.3±2.107 a
3.0	19.4	1.0	32.7	0.009±0.0019 b	0.826	69.9±1.073 b
3.0	38.8	1.0	32.7	0.025±0.0018 c	0.979	27.9±0.436 c
3.0	77.6	1.0	32.7	0.016±0.0023 d	0.921	44.8±0.435 d
**Variation of Fe^{2+} concentration**						
3.0	38.8	0.5	32.7	0.017±0.0019 a	0.952	40.3±0.573 a
3.0	38.8	1.0	32.7	0.025±0.0018 b	0.979	27.9±0.436 b
3.0	38.8	2.5	32.7	0.017±0.0025 a	0.921	42.3±1.862 a
3.0	38.8	5.0	32.7	0.010±0.0024 c	0.799	59.9±0.551 c
**Variation of irradiance intensity**						
3.0	38.8	1.0	0.0	0.006±0.0017 a	0.875	117.2±3.676 a
3.0	38.8	1.0	5.2	0.008±0.0019 b	0.816	86.6±1.140 b
3.0	38.8	1.0	18.3	0.011±0.0023 c	0.852	51.1±0.899 c
3.0	38.8	1.0	32.7	0.025±0.0018 d	0.979	27.9±0.436 d

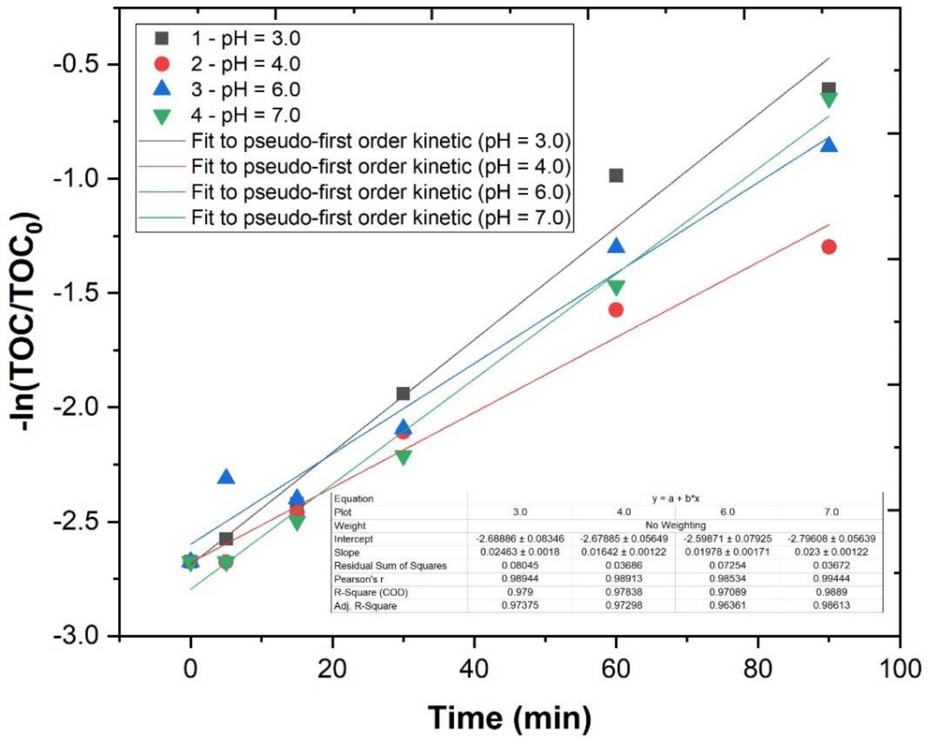

Figure S1. Pseudo-first order kinetic rate along the photo-Fenton process at different pH (3-7) in UV-A-Fenton process. Experimental conditions: $[H_2O_2]$ = 38.8 mM, $[Fe^{2+}]$ = 1.0 mM, agitation 350 rpm, T = 25°C, radiation UV-A, I_{UV} = 32.7 Wm^{-2}, t = 90 min.

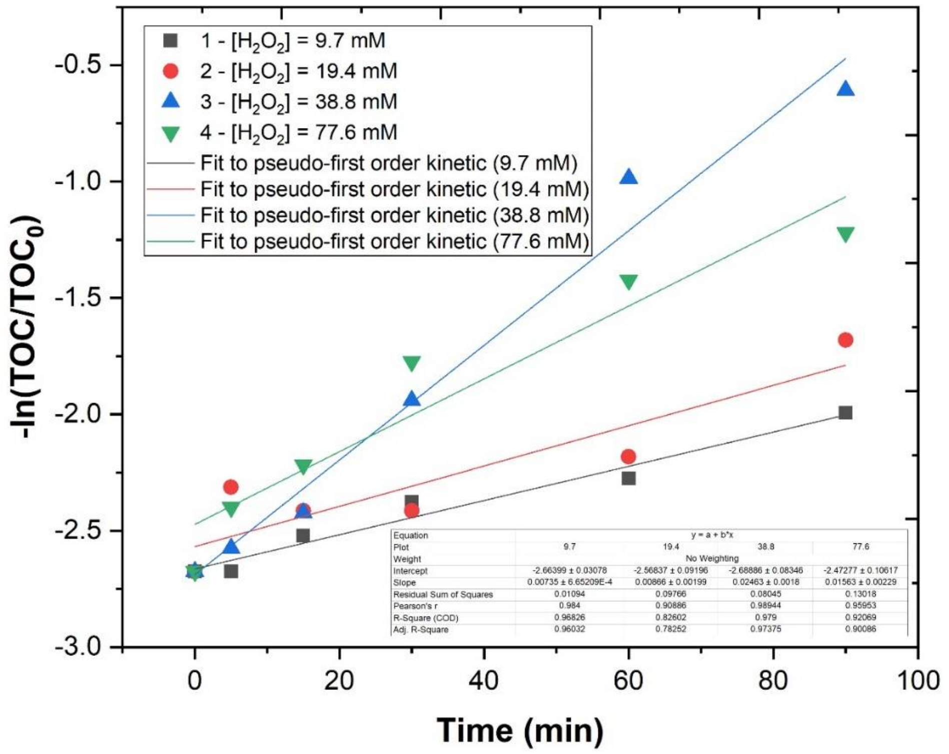

Figure S2. Pseudo-first order kinetic rate along the photo-Fenton process at different H_2O_2 concentrations (9.7 – 77.6 mM) in UV-A-Fenton process. Experimental conditions: $[Fe^{2+}]$ = 1.0 mM, pH = 3.0, agitation 350 rpm, T = 25°C, radiation UV-A, I_{UV} = 32.7 Wm^{-2}, t = 90 min.

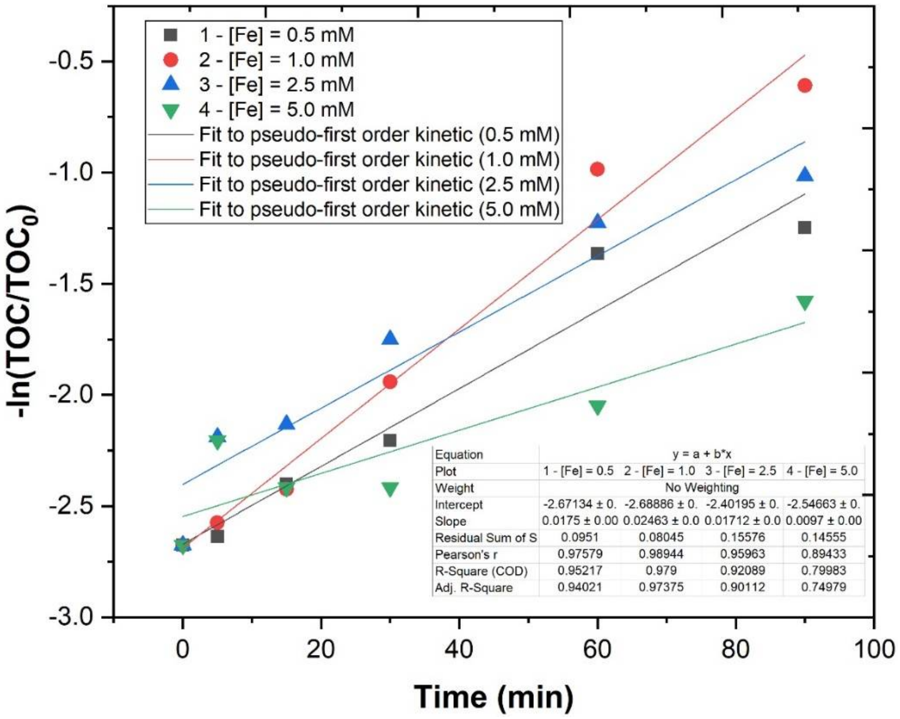

Figure S3 Pseudo-first order kinetic rate along the photo-Fenton process at different Fe^{2+} concentrations (0.5 – 5.0 mM) in UV-A-Fenton process. Experimental conditions: $[H_2O_2]$ = 38.8 mM, pH = 3.0, agitation 350 rpm, T = 25°C, radiation UV-A, I_{UV} = 32.7 Wm^{-2}, t = 90 min.

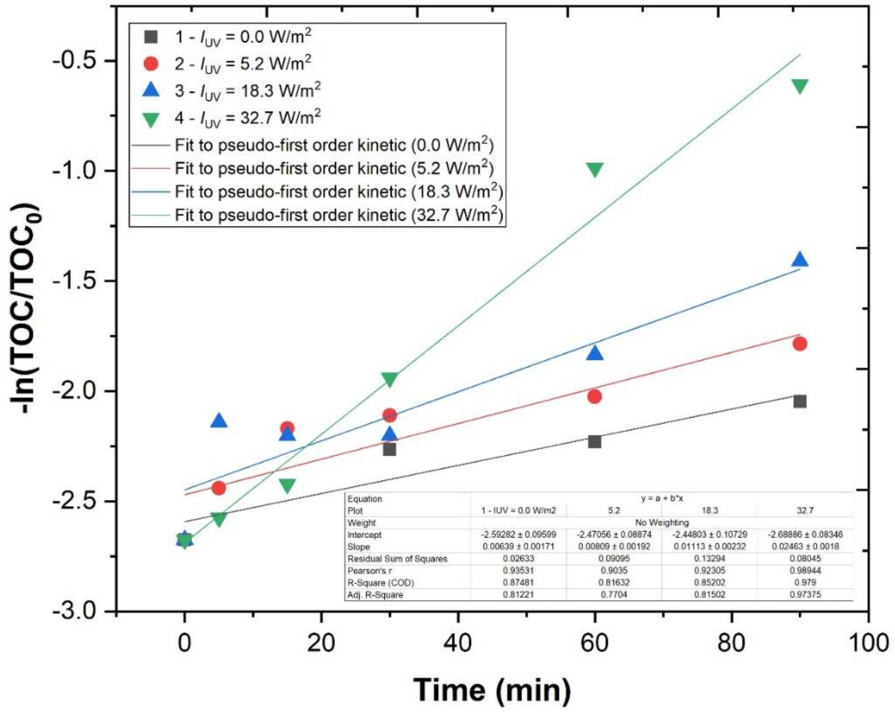

Figure S4 Pseudo-first order kinetic rate along the photo-Fenton process at different UV-A radiation intensity (0.0 – 32.7 W/m^{-2}) in UV-A-Fenton process. Experimental conditions: $[H_2O_2]$ = 38.8 mM, $[Fe^{2+}]$ = 1.0 mM, pH = 3.0, agitation 350 rpm, T = 25°C, t = 90 min.
